# Management of Patients With Unexplained Syncope and Bundle Branch Block: Predictive Factors of Recurrent Syncope

**DOI:** 10.7759/cureus.35827

**Published:** 2023-03-06

**Authors:** Ioannis Doundoulakis, Dimitris Tsiachris, Athanasios Kordalis, Stergios Soulaidopoulos, Petros Arsenos, Anastasia Xintarakou, Leonidas Koliastasis, Panayotis K Vlachakis, Konstantinos Tsioufis, Konstantinos A Gatzoulis

**Affiliations:** 1 First Department of Cardiology, National and Kapodistrian University, “Hippokration” Hospital, Athens, Athens, GRC

**Keywords:** bifascicular block, bundle branch block, electrophysiology study, pacemaker, syncope

## Abstract

Syncope in patients with bundle branch block (BBB) is often due to advanced atrioventricular (AV) block. The objective of the present “real-world” study was to evaluate the optimal management in patients with unexplained syncope and BBB and to identify factors that predict the recurrence of syncope. This is a single-center observational prospective registry of 131 consecutive patients undergoing invasive electrophysiology study (EPS) for recurrent unexplained presyncope or syncope attacks and BBB. When the EPS-derived diagnosis was reached, a decision to proceed with a permanent pacemaker was offered to the patient. An implantable loop recorder was inserted in the rest of the population. A total of 131 consecutive patients with unexplained syncope and BBB (67.2% male; age 63.7 ± 16.5 years) underwent EPS during the study period. The distribution of conduction disturbance patterns was as follows: isolated left bundle branch block (LBBB): 23.7%; LBBB with first AV block: 8.4%; isolated right bundle branch block (RBBB): 10.7%; RBBB with first AV block: 8.4%; isolated left anterior/posterior fascicular block: 13%; left anterior/posterior fascicular block with first AV block: 5.3%; isolated bifascicular block: 16.8%; and bifascicular block with first AV block: 13.7%. In the multivariate analysis, the only predictors of recurrent syncope were bifascicular block (hazard ratio (HR): 4.16, 95% confidence interval (CI): 1.29, 13.41, P: 0.017) and HV interval ≥ 60 msec (HR: 3.58, 95% CI: 1.12, 11.46, P: 0.032). An EPS-based strategy identifies a subset of patients who will benefit from permanent pacing. HV interval ≥ 60 msec and the presence of a bifascicular block were strongly related to syncope recurrence.

## Introduction

Syncope in patients with bundle branch block (BBB) is often due to advanced atrioventricular block (AVB) [1]. However, other mechanisms may also be involved in patients with syncope whose cause remains unknown after an initial assessment [2,3]. Independently from the underlying cause, it is crucial to identify patients at the highest risk who may require a pacemaker [4].

On the one hand, with respect to bifascicular blocks, both the European Society of Cardiology (ESC) and the American College of Cardiology/American Heart Association Task Force on Clinical Practice Guidelines along with the Heart Rhythm Society (ACC/AHA/HRS) guidelines recommend an electrophysiology study (EPS) to be considered (class IIa recommendation, level B evidence) in the diagnostic work-up of patients with unexplained syncope. If EPS is positive, implantation of a pacemaker system is indicated (class I recommendation, level B evidence) [5,6]. On the other hand, we still lack clear answers to seemingly simple questions, including the appropriate use of EPS and the exact criteria for pacing based on EPS measurements in patients with BBB without bifascicular block [7]. Previous important studies have demonstrated that the EPS-guided strategy is generally safe [2,8,9]. Apart from that, there is mounting epidemiological evidence suggesting that beyond the presence of an increased risk of syncope and complete heart block among patients with higher degrees of fascicular blocks, a substantial association of apparently less complex fascicular blocks with the development of high degree AVB also exists [10].

In this context, the objective of the present “real-world” study was to evaluate the optimal management in patients with unexplained syncope and BBB and to identify factors that predict the recurrence of syncope. This article was previously presented as a meeting abstract at the International Congress of Cardiology on October 20, 2022.

## Materials and methods

This is a single-center observational prospective registry of 131 consecutive patients undergoing invasive EPS for recurrent unexplained presyncope or syncope attacks and BBB at the First University Department of Cardiology in the “Hippokration” Hospital between January 2000 and December 2017.

All patients had undergone appropriate cardiologic (including at least 24 hours of ambulatory monitoring) and neurological evaluation, without reaching a diagnosis as to the cause of syncope. Patients with an established indication for pacing (third-degree AVB, Mobitz II, or advanced second-degree AVB) or with severely depressed left ventricular ejection fraction (<35%) were excluded. The protocol of the study was approved by our Research Ethical Committee and informed written consent was taken from all patients after explaining the pros and cons of the EPS.

Electrocardiography and EPS findings

The methodology of obtaining the non-invasive electrocardiogram (ECG) features and the invasive EPS results have been previously published and have been consistently performed since the beginning of the study [4,11-13]. EPS was considered positive when any of the following was detected:

1) Basic HV interval of ≥ 60 msec

2) Wenckebach cycle length ≥ 500 msec and 2:1 AVB cycle length ≥ 400 msec

3) Effective refractory period of the atrioventricular node ≥ 450 msec

All patients underwent also sinus node function assessment in order to detect the concurrent presence of sinus node disease:

1) Corrected Sinus Node Recovery Time (CSNRT) ≥ 525 msec (any sinoatrial conduction time ≥ 140 msec or chronotropic response to atropine ≤ 90 bpm were identified and noticed)

The EPS protocol was completed with right ventricular stimulation (from two sites, ≤3 premature extra stimuli and at 2 cycle lengths) generally before and after isoproterenol infusion was delivered as well as carotid sinus massage according to the degree of clinical suspicion for an associated electrical disturbance. When the EPS-derived diagnosis was reached, and in relation to other clinical laboratory features present, a decision to proceed with a permanent pacemaker was offered to the patient. An implantable loop recorder was offered to the rest of the population if they were available in the center (Initiation enrolment of the study: 2000). The study outcome measure was the time to the event of recurrence syncope (syncope-free survival).

Statistical analysis

Continuous variables were summarized with mean and standard deviation (SD) and compared using independent samples t-test or Mann Whitney U test as appropriate. Categorical variables were described with frequencies and percentages and compared using the Chi-square test. Kaplan-Meier curves were used to visualize survival free from syncope occurrence and the log-rank test was applied to assess the presence of statistically significant differences according to pacemaker status. Cox regression was used in order to assess and compare the impact of parameters on syncope recurrence in patients without ABP. The significance level was set to p-value < 0.05 and two-tailed. Data analysis was performed using the IBM/Statistical Package for Social Sciences (SPSS version 24, IBM, Armonk, NY) program.

## Results

A total of 131 consecutive patients with unexplained syncope and BBB (67.2% male; age 63.7 ± 16.5 years) underwent EPS during the study period. The population characteristics, comorbidities, and ECG abnormalities are listed in Table 1. The distribution of conduction disturbance patterns was as follows: isolated left bundle branch block (LBBB): 23.7%; LBBB with first AV block: 8.4%; isolated right bundle branch block (RBBB): 10.7%; RBBB with first AV block: 8.4%; isolated left anterior/posterior fascicular block: 13%; left anterior/posterior fascicular block with first AV block: 5.3%; isolated bifascicular block: 16.8%; and bifascicular block with first AV block: 13.7%.

**Table 1:** Baseline patients’ characteristics LVEF: left ventricular ejection fraction; ECG: electrocardiogram; AV: atrioventricular

Variables	Overall (N=131)	Pacemaker (N=86)	No Pacemaker with Positive Diagnostic Workup (N=27)	No Pacemaker with Negative Diagnostic Workup (N=18)
Age (years)	63.7 (±16.5)	67.2 (±11.4)	60.4 (±22.0)	52.0 (±22.2)
LVEF (%)	55.4 (±12.2)	53.5 (±12.8)	57.4 (±10.3)	60.8 (±9.7)
Sex (Male)	88 (67.2)	57 (66.3)	19 (70.4)	12 (66.7)
Presyncope (N of patients) (%)	44 (33.6)	31 (36.0)	8 (29.6)	5 (27.8)
Syncope (N of patients) (%)	97 (74.0)	64 (74.4)	20 (74.1)	13 (72.2)
Presyncope (N of events)	2.0 (±1.6)	2.0 (±1.1)	2.8 (±3.0)	1.0 (±0.0)
Syncope (N of events)	1.9 (±1.0)	2.0 (±1.0)	2.3 (±1.1)	1.2 (±0.4)
Hypersensitive Carotid Sinus Syndrome	5 (4.2)	1 (1.1)	2 (7.4)	0 (0.0)
Follow-up (months)	45.6 (±27.7)	48.1 (±30.5)	36.8 (±19.7)	46.9 (±21.2)
Organic heart disease				
Coronary artery disease (%)	29 (22.3)	25 (29.1)	3 (11.5)	1 (5.6)
Hypertrophic cardiomyopathy (%)	3 (2.3)	2 (2.3)	0 (0.0)	1 (5.6)
Dilated cardiomyopathy (%)	13 (10.0)	10 (11.6)	2 (7.4)	1 (5.6)
Valvular heart disease (%)	12 (9.2)	6 (7.0)	1 (3.7)	5 (27.8)
12-lead ECG				
Heart rate	64.2 (±9.7)	62.8 (±62.9)	64.3 (±8.0)	70.8 (±12.7)
First-degree atrioventricular block (PR ≥200 msec) (%)	47 (35.8)	41 (47.7)	6 (22.2)	0 (0.0)
Isolated Right Bundle Branch Block (%)	14 (10.7)	3 (3.5)	4 (14.8)	7 (38.9)
Right Bundle Branch Block with first AV block (%)	11 (8.4)	9 (10.5)	2 (7.4)	0 (0.0)
Isolated Left Bundle Branch Block (%)	31 (23.7)	24 (27.9)	4 (14.8)	3 (16.7)
Left Bundle Branch Block with first AV block (%)	11 (8.4)	9 (10.5)	2 (7.4)	0 (0.0)
Isolated Left Hemiblock (%)	17 (13)	3 (3.5)	8 (29.6)	6 (33.3)
Left Hemiblock with first AV block (%)	7 (5.3)	7 (8.1)	0 (0.0)	0 (0.0)
Bifascicular block (%)	22 (16.8)	15 (17.4)	5 (18.5)	2 (11.1)
Bifascicular block with first AV block (%)	18 (13.7)	16 (18.6)	2 (7.4)	0 (0.0)
Holter monitoring				
Mean 24-hour heart rate £ 60 bpm (%)	19 (14.8)	15 (17.9)	4 (15.4)	0 (0.0)
Sinus pauses ³ 2 sec (%)	14 (10.8)	12 (14.1)	1 (3.7)	1 (5.6)
Second-degree atrioventricular block (%)	15 (11.6)	14 (16.3)	1 (3.7)	1 (5.6)

The EPS results are presented in Table 2. The option of a permanent anti-bradycardia pacemaker (ABP) was offered to 111 patients according to the results of the EPS. Since the comprehensive diagnostic workup failed to reveal the origin of recurrent syncope in 20 patients, the implantation of a loop recorder was available and offered in 10 of them. After the completion of the process, ABP was offered to another two patients. Eighty-six patients received the ABP, while 27 declined. In all of these patients, pacemaker indication was due to AV node conduction disease. Any patient with coexisting prolonged sinus node recovery time also received a pacemaker.

**Table 2:** Results of the electrophysiology study

Variables	Overall (N=131)	Pacemaker (N=86)	No Pacemaker with Positive Diagnostic Workup (N=27)	No Pacemaker with Negative Diagnostic Workup (N=18)
HV interval (msec)	61.6 ± 23.1	68.2 ± 24.1	53.6 ± 16.3	42.4 ± 7.4
HV interval ≥60 msec (%)	73 (55.7)	62 (72.1)	11 (40.7)	0 (0.0)
Wenckebach cycle length (msec)	437.4 ± 113.0	465.3 ± 124.5	402.63 ± 65.2	361.1 ± 44.7
Wenckebach cycle length ≥500 msec (%)	29 (22.5)	26 (31.0)	3 (11.1)	0 (0.0)
2:1 atrioventricular block cycle length (msec)	376.4 ± 86.7	397.9 ± 94.4	354.2 ± 55.6	309.2 ± 27.8
2:1 atrioventricular block cycle length ≥400 msec (%)	36 (27.9)	28 (32.9)	8 (29.6)	0 (0.0)
Effective refractory period of the atrioventricular node (msec)	351.2 ± 97.3	381.5 ± 106.3	330.0 ± 72.2	280.0 ± 49.8
Effective refractory period of the atrioventricular node ≥450 msec (%)	7 (5.3)	5 (5.8)	2 (7.4)	0 (0.0)
Sinus node disease (%)	35 (26.9)	29 (34.1)	6 (22.2)	0 (0.0)

Follow-up

**Figure 1:**
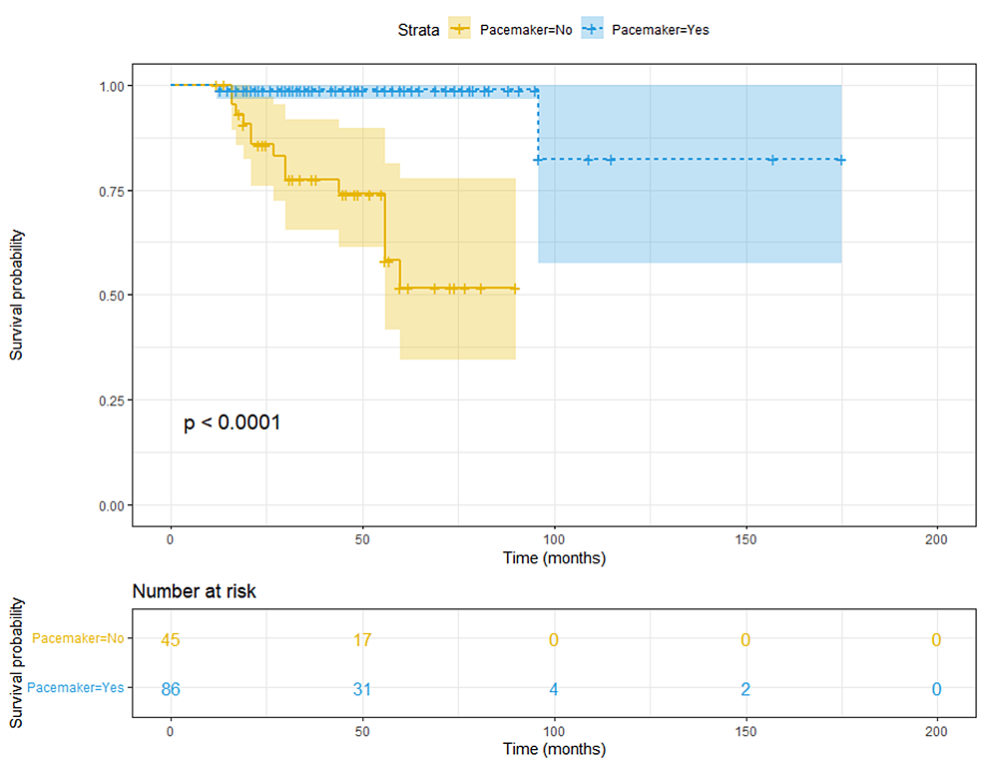
Kaplan-Meier curves for syncope-free survival according to permanent pacing status

Over a mean follow-up of approximately four years (46.4 ± 27.5 months), the primary outcome event (syncope) occurred in 16 of 131 patients (12.2%), two of 86 (2.3%) in the ABP group as compared to 11 of 27 (40.7%) in the no pacemaker group with positive diagnostic workup and three of 18 (16.7%) in the no pacemaker group with negative diagnostic workup (Table 3). In the Kaplan-Meier analysis, time-to-syncope recurrence was significantly longer for the pacemaker-implanted patients (p<0.001, Figure 1).

**Table 3:** Syncope recurrence rates according to the type of the bundle branch block EPS: electrophysiology study; AV: atrioventricular

Type of the Bundle Branch Block	Prevalence (%)	Positive EPS (%)	Pacemaker	Syncope Recurrence
Isolated Right Bundle Branch Block	14 (10.7)	7 (50.0)	No: 4	1
			Yes: 3	1
Right Bundle Branch Block with first AV block	11 (8.4)	11 (100)	No: 2	1
			Yes: 9	0
Isolated Left Bundle Branch Block	31 (23.7)	28 (90.3)	No: 4	1
			Yes: 24	1
Left Bundle Branch Block with first AV block	11 (8.4)	11 (100)	No: 2	0
			Yes: 9	0
Isolated Left Hemiblock	17 (13)	9 (52.9)	No: 7	3
			Yes: 0	0
Left Hemiblock with first AV block	7 (5.3)	7 (100)	No: 0	0
			Yes: 7	0
Bifascicular block	22 (16.8)	20 (90.9)	No: 5	4
			Yes: 15	0
Bifascicular block with first AV block	18 (13.7)	18 (100)	No: 2	1
			Yes: 15	0

Predictors of recurrent syncope in patients with unexplained syncope and BBB

Cox regression was used in order to assess and compare the impact of parameters on syncope recurrence in patients without ABP (N=45). The pattern of BBB was a predictor of recurrent syncope in the univariate analysis: isolated RBBB (hazard ratio (HR): 0.17, 95% confidence interval (CI): 0.02, 1.31, P: 0.089) is a significant predictor for being at low risk for syncope recurrence, while bifascicular block is a significant predictor for being at high risk (HR: 7.13, 95% CI: 2.42, 21.01, P: 0.001). The following parameter of the EP test were also predictors of recurrent syncope during follow-up: HV interval ≥ 60msec (HR: 5.59, 95% CI: 1.93, 16.20, P: 0.002). In the multivariate analysis, the only predictors were bifascicular block (HR: 4.16, 95% CI: 1.29, 13.41, P: 0.017) and HV interval ≥ 60msec (HR: 3.58, 95% CI: 1.12, 11.46, P: 0.032). Univariate and multivariate predictors are listed in Table 4.

**Table 4:** Cox regression model for recurrent syncope in patients without a pacemaker OR: odds ratio, CI: confidence intervals; AV: atrioventricular ^a ^With backward elimination according to likelihood ratio criteria

	Univariate			Multivariate^a^		
Variables	OR	95% CI	P-value	OR	95% CI	P-value
Age	1.00	0.98, 1.03	0.852			
Ejection fraction	1.00	0.94, 1.06	0.963			
Organic heart disease	0.91	0.25, 3.27	0.881			
First-degree atrioventricular block	1.10	0.24, 4.73	0.943			
Isolated Right Bundle Branch Block	0.17	0.02, 1.31	0.089	0.33	0.04, 2.76	0.308
Right Bundle Branch Block with first AV block	1.49	0.19, 11.43	0.704			
Isolated Left Bundle Branch Block	0.81	0.18, 3.62	0.778			
Left Bundle Branch Block with first AV block	0.05	0.01, 1706.77	0.566			
Isolated Left Hemiblock	0.67	0.18, 2.46	0.549			
Left Hemiblock with first AV block	N/A	N/A	N/A			
Bifascicular block	7.13	2.42, 21.01	0.001	4.16	1.29, 13.41	0.017
Bifascicular block with first AV block	2.11	0.27, 16.39	0.476			
Sinus node disease	0.87	0.19, 3.89	0.852			
HV interval ≥60msec	5.59	1.93, 16.20	0.002	3.58	1.12, 11.46	0.032
HV interval ≥70msec	2.33	0.64, 8.52	0.201			
Wenckebach cycle length ≥500 msec	1.12	0.14, 8.70	0.913			
2:1 atrioventricular block cycle length ≥400 msec	1.73	0.53, 5.64	0.363			
Effective refractory period of the atrioventricular node ≥450 msec	1.19	0.26, 15.50	0.510			

## Discussion

In this analysis of “real-world” data in patients with a history of unexplained syncope and BBB, an EPS-based strategy was shown to be adequately efficient in identifying a sample of patients who will benefit from permanent pacing, in terms of preventing new syncopal episodes. This study analyzed the predictive parameters of syncope recurrence and displayed that only an HV interval ≥ 60msec and bifascicular block are strong independent predictive factors.

Syncope in patients with BBB is often due to advanced AVB [14], especially in those with prolonged HV intervals. In our real-world study, after a negative EPS study, only 16.7% of patients presented again with a syncopal episode. The proposed HV interval prolongation has remarkably changed from as long as 100 msec in the previous to as short as 70 msec in the current European guidelines [5,15] while it is not fully defined in the current American guidelines [6]. Recent data from other studies showed that an HV interval ≥ 60 msec was a predictor of pacing [2,16]. In this way, the use of 60 msec as the gold standard cut-off to implant an ABP in patients with syncope and BBB may improve the EPS-guided strategy for these high-risk patients.

Among all parameters, only the bifascicular block and the HV interval were found to be independent predictors for syncope recurrence in the multivariate cox regression analysis. A study with long-term follow-up in patients with bifascicular block and unexplained syncope showed that an EPS-guided approach is the most effective way of identifying the appropriate candidates for pacing [17]. Similar results emerged from another study in the targeted population of our study (syncope and BBB) [2]. The authors of this study proposed a simple risk score with bifascicular block and HV interval which is also effective in our population. However not infrequently multiple causes of syncope are revealed during a comprehensive approach [11,18,19]. Failure to define the cause of recurrent syncope despite analytical invasive and noninvasive investigation affects not only the quality of life but also the patient’s prognosis [20]. In such cases, an implantable loop may be the most appropriate treatment plan [21].

This is an observational study. The main limitation is the non-randomized nature of the permanent pacemaker implantation, introducing a potential for bias. Patients with a syncope potential suggestive of noncardiac syncope may be not included in this sample size, because only patients referred for EPS were included. In this way, a high percentage of patients with pathological findings in the EP study is observed in our cohort. In addition to this, apart from a small number of patients, the HV interval did not measure during class I drug administration. Implantable loop recorders were offered only in 10 patients, as a result of the initiation of the study before the release of these devices. However, the diagnostic approach is strict and reproducible in our center [4,12]. Finally, the small number of no-pacemaker patients may criticize the results from the multivariate cox regression analysis.

## Conclusions

In our population of patients with syncope and BBB, an EPS-based strategy identifies a subset of patients who will benefit from permanent pacing, in terms of preventing new syncopal episodes. HV interval ≥ 60 msec and the presence of a bifascicular block were strongly related to syncope recurrence. Our study supports previous studies' findings, in which the cut-off for the HV interval in patients with syncope and BBB was defined at 60 msec. The upcoming guidelines may examine the use of 60 msec as the cutoff value in considering EPS as positive for pacemaker implantation, especially in patients with bifascicular block.
